# An analysis of the physicochemical properties of oral drugs from 2000 to 2022†

**DOI:** 10.1039/d4md00160e

**Published:** 2024-07-22

**Authors:** Rachael Pirie, Harriet A. Stanway-Gordon, Hannah L. Stewart, Kirsty L. Wilson, Summer Patton, Jack Tyerman, Daniel J. Cole, Katherine Fowler, Michael J. Waring

**Affiliations:** a Chemistry, School of Natural and Environmental Sciences, Newcastle University Bedson Building Newcastle upon Tyne NE1 7RU UK; b Cancer Research Horizons Newcastle Drug Discovery Unit, Chemistry, School of Natural and Environmental Sciences, Newcastle University Bedson Building Newcastle upon Tyne NE1 7RU UK mike.waring@ncl.ac.uk; c Cancer Research Horizons Therapeutic Innovation, Jonas Webb Building Babraham Research Campus Cambridge CB22 3AT UK

## Abstract

Calculable physicochemical descriptors are a useful guide to assist compound design in medicinal chemistry. It is well established that controlling size, lipophilicity, hydrogen bonding, flexibility and shape, guided by descriptors that approximate to these properties, can greatly increase the chances of successful drug discovery. Many therapeutic targets and new modalities are incompatible with the optimal ranges of these properties and thus there is much interest in approaches to find oral drug candidates outside of this space. These considerations have been a focus for a while and hence we analysed the physicochemical properties of oral drugs approved by the FDA from 2000 to 2022 to assess if such concepts had influenced the output of the drug-discovery community. Our findings show that it is possible to find drug molecules that lie outside of the optimal descriptor ranges and that large molecules in particular (molecular weight >500 Da) can be oral drugs. The analysis suggests that this is more likely if lipophilicity, hydrogen bonding and flexibility are controlled. Crude physicochemical descriptors are useful in that regard but more accurate and robust means of understanding substructural classes, shape and conformation are likely to be required to improve the chances of success in this space.

## Abstract 2

Calculable physicochemical descriptors are a useful guide to assist compound design in medicinal chemistry.

## Graphical abstract

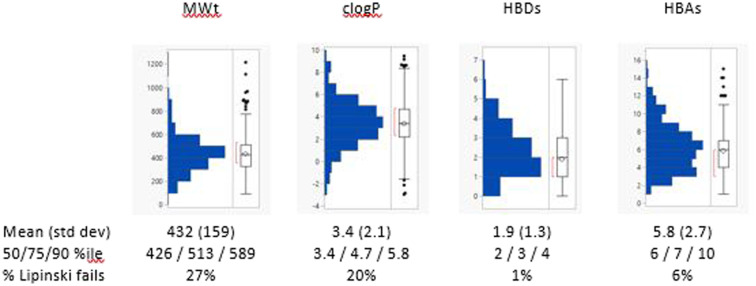

## Introduction

Control of physicochemical properties, perhaps most notably size, lipophilicity and hydrogen bonding is well established to be critical to achieving acceptable ADMET properties, in particular those relating to desirable oral pharmacokinetics. To guide compound design, these complex parameters can be quantified to a reasonable degree by simple numerical descriptors such as molecular weight (MWt) for size, calculated lipophilicity (clog *P*) for lipophilicity and counts of hydrogen bond donors (HBD) and acceptors (HBA) for hydrogen bonding.^1–6^ Desirable ranges for these and related parameters in which the probability of achieving desirable ADMET have been defined in various ways, with “Lipinski's Rule of 5” being the best known and adopted. This rule states that oral absorption is less likely for compounds that have two or more of MWt >500, clog *P* >5, HBD >5 and HBA >10.

Whilst the objective of achieving orally bioavailable drugs means the majority of drug discovery projects aim to operate within Lipinski space, achieving potent compounds with such properties generally requires compounds to bind within a defined pocket in the protein target, which is hydrophobic in nature but also has the potential to form productive polar interactions with a ligand.^7^ Proteins that do not possess such features are challenging (in extreme cases considered intractable)^8^ and the desire to drug such proteins has prompted a great deal of interest in identifying compounds that have desirable ADMET properties but do not meet Lipinski criteria – generally referred to as “Beyond rule of 5”. The importance of this area has been further heightened by the recent interest in chimeric molecules such as heterobifunctional degraders, which necessitate high MWt compounds.^9–11^ At the same time, the importance of MWt as an indicator of drug like properties has been questioned based on an analysis of approved drugs up until 2017.^12^

As a consequence, there has been significant recent interest in defining the types of molecules and their related properties that can achieve oral bioavailability outside of Lipinski space.^13–16^ This has included interest in specific structural features, in particular macrocycles that are postulated to permit higher MWt, and in the definition of new descriptor-based rules.

Sufficient time has elapsed since the renewed interest in Beyond rule of 5 design that, if justified, there would be an observable effect on the properties of drugs emerging recently. To investigate this, we carried out an analysis of the properties of FDA drugs approved during the period 2000 to 2022.

## Results and discussion

Oral drug approvals from 2000 to 2022 inclusive were obtained from the FDA website.^17^ Calculated properties for each compound were generated using RDKit.

There were 382 compounds approved during the selected period. Cancer was the most frequent major disease indication (*n* = 95) followed by nervous system (*n* = 87) then infection (*n* = 71).

There were 40, 34 and 21 approvals in GI/metabolism, cardiovascular and respiratory/inflammation, respectively.

Calculated property distributions of the 382 compounds were analysed. There were 10 compounds that appeared as outliers and are known to act in the gastrointestinal tract, being well understood not to be absorbed or that represented combinations of older drugs, which were excluded from the subsequent analysis. A further compound (ixazomib) did not generate calculated data, presumably due to the presence of the boronic acid functionality. The remaining 371 compounds had a mean MWt of 432, clog *P* of 3.4, 2 HDB and 6 HBA (Table 1). Lipinski's original limits were based on the 90th percentile of each of these descriptors; if they had been derived from this dataset, the rules would be MWt < 589 Da, clog *P* < 5.8, HBD < 4 and HBA < 10.

**Table 1:** Physicochemical property distributions of FDA approved oral drugs from 2000–2022 (*n* = 371)

	MWt	clog *P*	HBDs	HBAs
	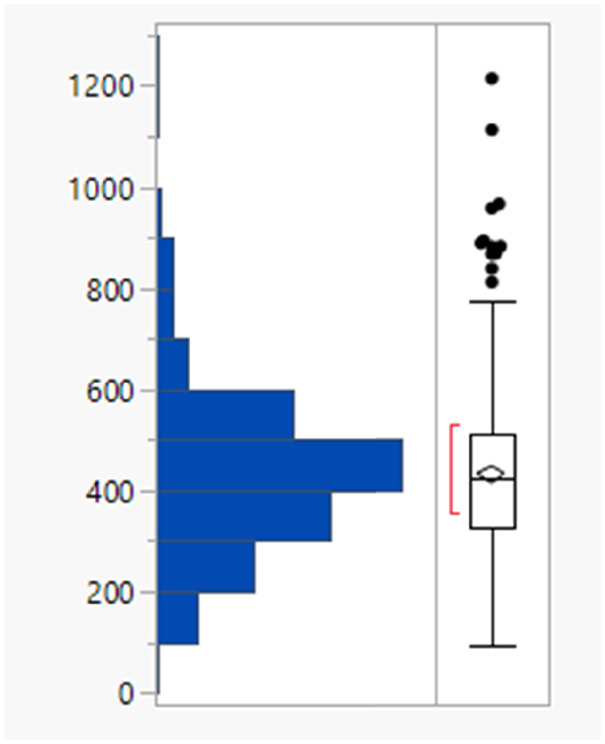	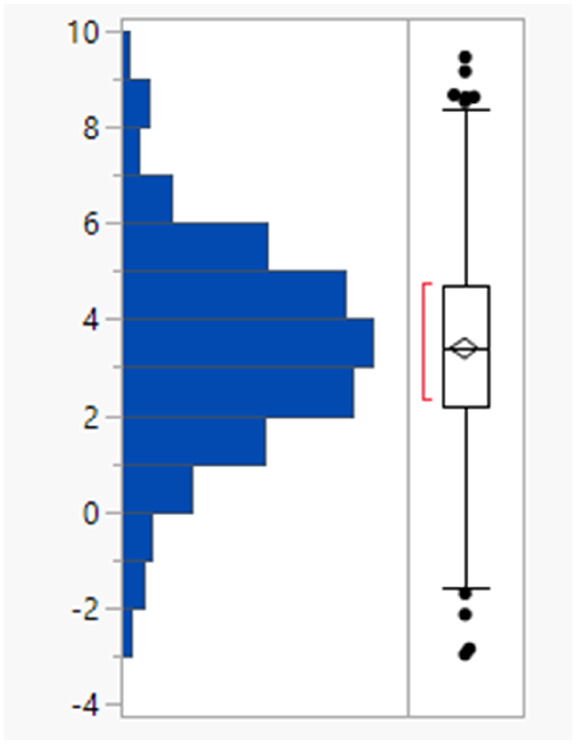	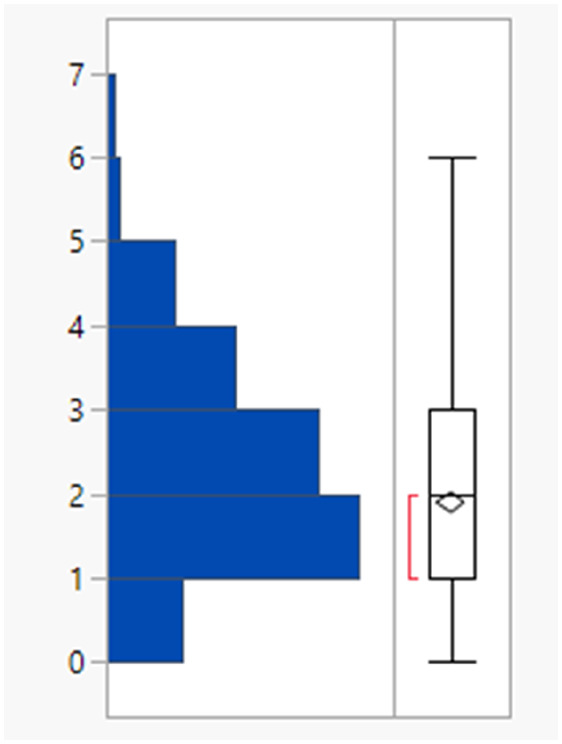	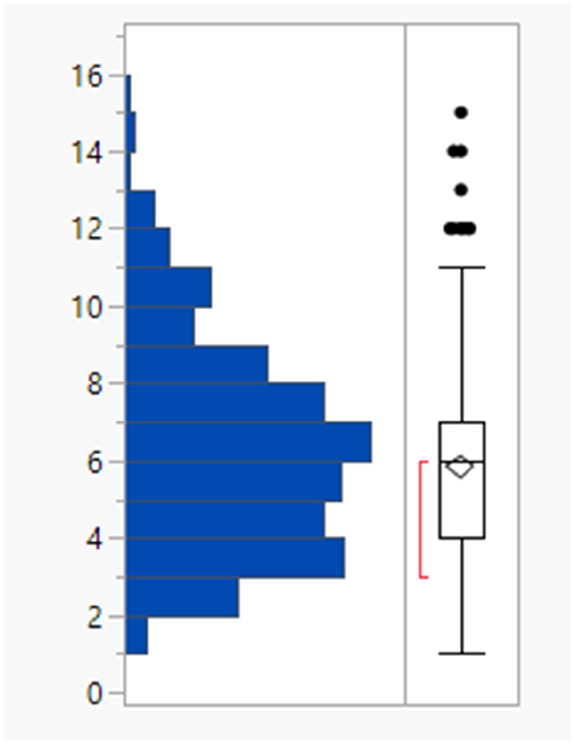
Mean (std dev)	432 (159)	3.4 (2.1)	1.9 (1.3)	5.8 (2.7)
50/75/90 %ile	426/513/589	3.4/4.7/5.8	2/3/4	6/7/10
% Lipinski fails	27%	20%	1%	6%

The four Lipinski descriptors were not tightly correlated for this dataset (closest correlation was between HBAs and MWt (*r*^2^ = 0.69) then MWt and clog *P* (*r*^2^ = 0.65), Table S1†).

**Fig. 1:**
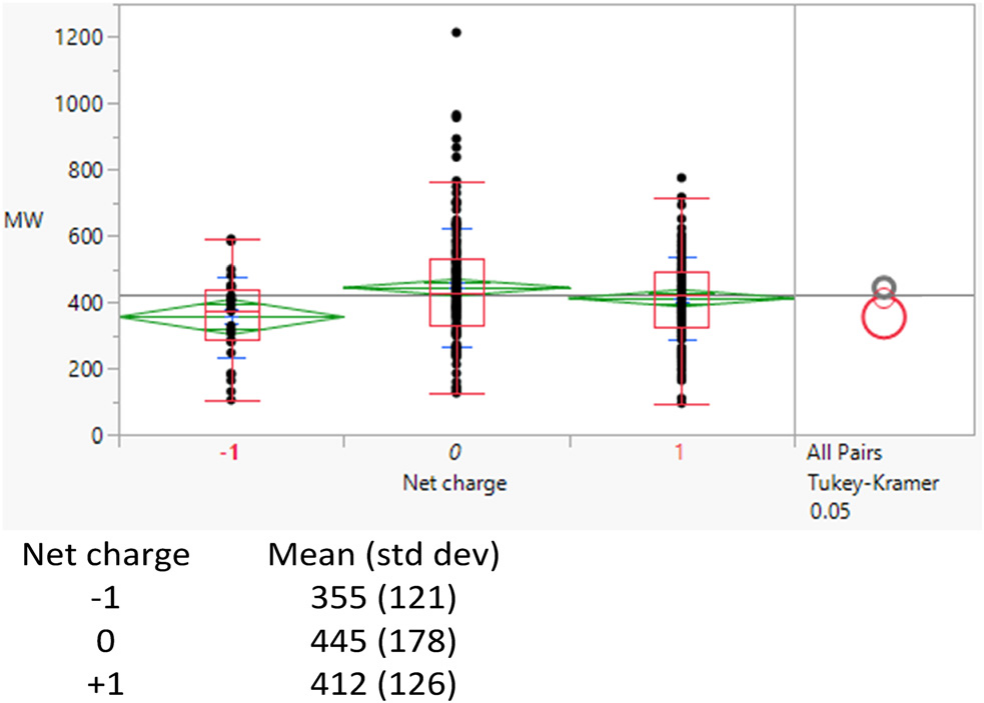
MWt distribution by ionisation type.

It might have been expected that higher clog *P* values arise from charged species, for which the corresponding log *D*_7.4_ values would be lower but there is no difference between the clog *P* distributions of the charged and uncharged compounds (Table S2†). The subset of monoacidic compounds (defined as those predicted to carry an overall net charge of −1 based on the sum of acidic and basic groups) had a significantly (Tukey–Kramer HSD test) lower mean MWt (355 Da) compared to the neutral set (overall charged groups sum to 0, 445 Da), presumably because acidic compounds are required to be smaller to achieve sufficient permeability (Fig. 1).^18^ Distributions of the HBD/HBAs were not significantly different between the ionisation types.

Within the dataset, there are perhaps a surprisingly large number of compounds that lie outside of Lipinski limits for any one of these descriptors, 27% of the compounds have MWt >500 Da and 20% have a clog *P* >5. HBD and HBA violations are less frequent (1.1% and 5.7% respectively).

Of course, Lipinski's rules actually state that poor oral bioavailability is likely if *two or more* of the rules are violated. In this dataset, 64 compounds (17%) violate two or more of the criteria. The proportion of Lipinski fails increased gradually over the period from 14 (12%) in 2000–2009 to 41 (20%) in 2010–2019 and with 15 (18%) in 2020–2022 (Fig. S1†), although this is against a background of an overall increase in the number of approvals over that period such that the proportion remains similar. There was a general increase in the individual parameters MWt, clog *P* and HBA over the period, whereas HBDs remained constant (Table S3†).

The 64 fails had a mean MWt of 656, mean clog *P* 5.7, mean HBD 2 and HBA 9 (Table 2). Comparing the Lipinski fails to the others in the set showed statistically significant differences in all descriptors, but HBD counts were far closer between the two (median and means both 2). All 64 compounds had MWt >500, 45 (70%) had clog *P* >5, 21 (33%) had HBA >10 but only 2 had HBD >5. The overall trends with HBD (fewer violations and similar distributions between the pass and fail set) is consistent with observations that limiting them is a key consideration in operating outside of Lipinski space.

**Table 2:** Physicochemical property distributions showing histograms of the subset of Lipinski fails 2000–2022 (*n* = 64) and one-way plots comparing them to the passes

	MWt	clog *P*
	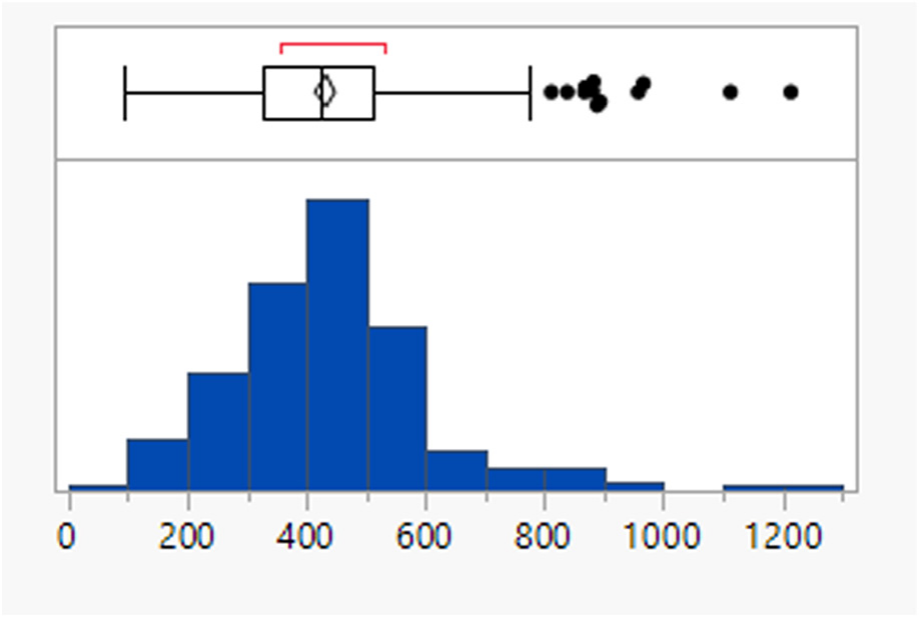	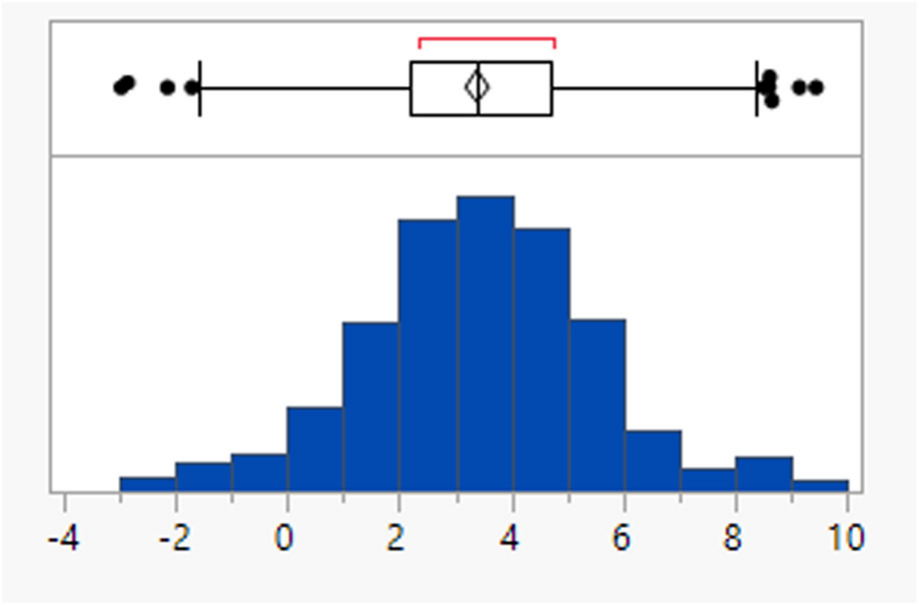
	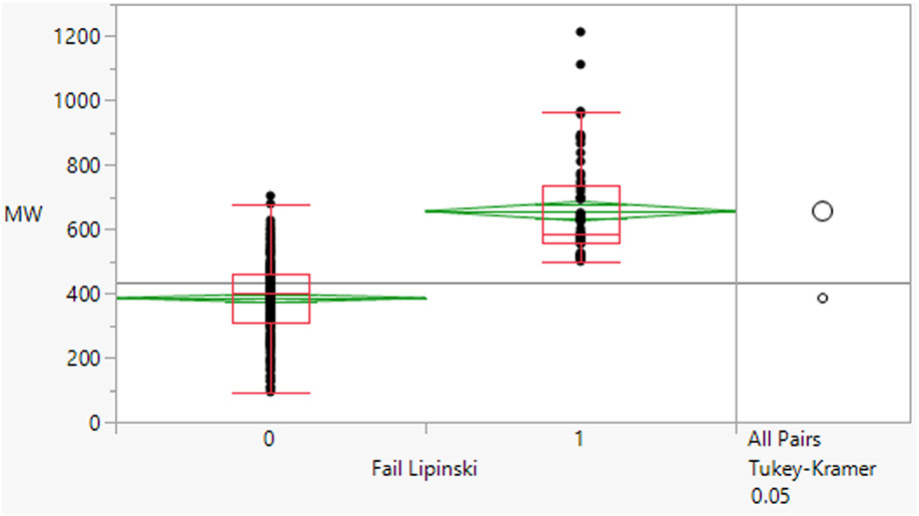	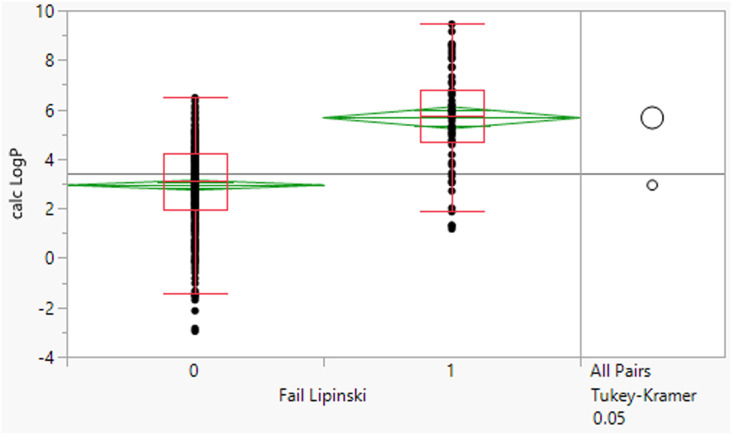
Mean	656	5.7
Median	583	5.7
	HBDs	HBAs
	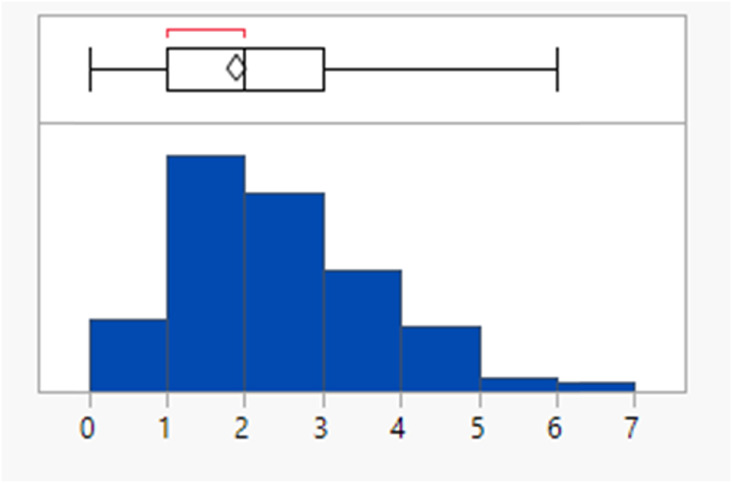	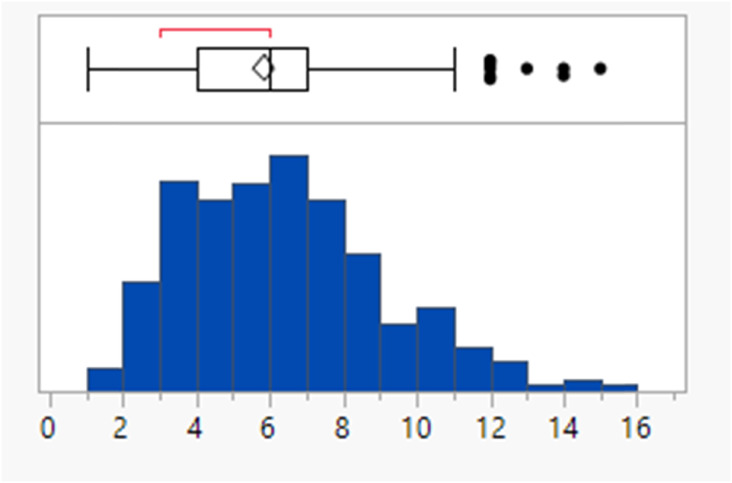
	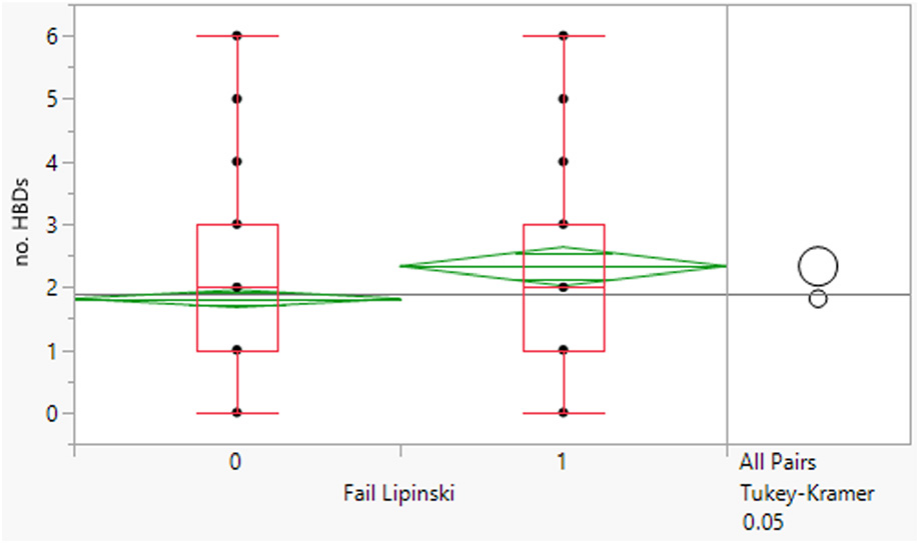	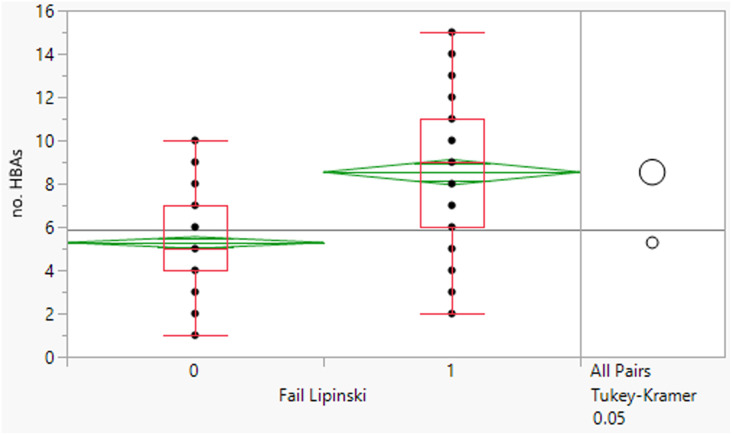
Mean	2	9
Median	2	9

**Fig. 2:**
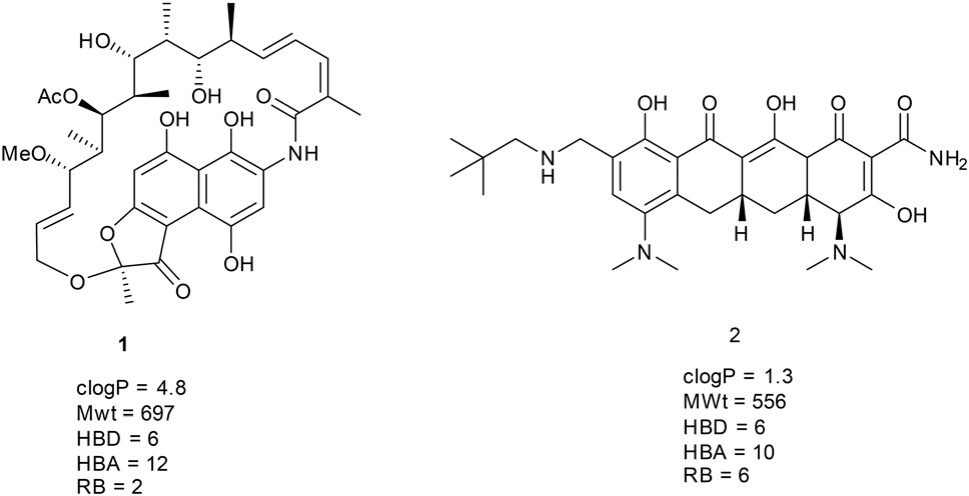
Structures of the two drugs failing Lipinski criteria with HBD >5.

Of the Lipinski fails, there are only two compounds that have >5 HBDs, rifamycin 1 (approved previously, but contained in new approval) and omadacycline 2 (Fig. 2). In both cases, there are apparent structural reasons why these compounds may behave differently, rifamycin is a macrocycle (see later) and omadacycline is a very rigid structure; in both cases, it is easy to conceive that the hydrogen bond donors can be satisfied by intramolecular hydrogen bonds and extensive intramolecular bonding is observed in small molecule crystal structures in both cases.^19,20^

The changes in property distributions are consistent with the idea that Lipinski descriptors are not wholly precise determinants of drug-likeness. Perhaps medicinal chemists are learning how to operate outside of Lipinski space as required to find drugs for more challenging targets and, hence, to work in property space in which achieving oral absorption is more challenging. The increase in numbers of approvals outside of Lipinski space over time is supportive of this idea. However, there is little general understanding of how to define areas of chemistry outside of Lipinski space that have increased chances of gaining drug-like properties.^11^ It would be expected that the chances of doing so will always be significantly lower than they are for compounds that are within it.

The increased MWt of the compounds in this dataset is perhaps the most striking deviation. The reason for high MWt compounds being disfavoured is primarily because larger molecules tend to be less permeable, in part because they are likely to contain more polar functionality and hydrogen bonding groups, but also because they have greater degrees of freedom in solution. Thus, larger molecules are likely to have higher enthalpic and entropic barriers to transition from aqueous solution to a phospholipid membrane. In this regard, MWt is a crude approximation of the size and shape of a molecule.

We considered whether the shape, flexibility and conformational profile of larger molecules are more relevant than MWt as determinants of drug-likeness. Such considerations are complex, and transcend the use of crude descriptors. Nevertheless, simple metrics that are approximations of shape and flexibility, such as rotatable bond count,^5^ aromatic ring count^21^ and fraction of sp^3^ atoms (Fsp^3^),^22^ have been adopted as measures of drug-likeness. Perhaps, if these were better determinants of drug-likeness, their distributions would look similar between the pass and fail subsets.

**Fig. 3:**
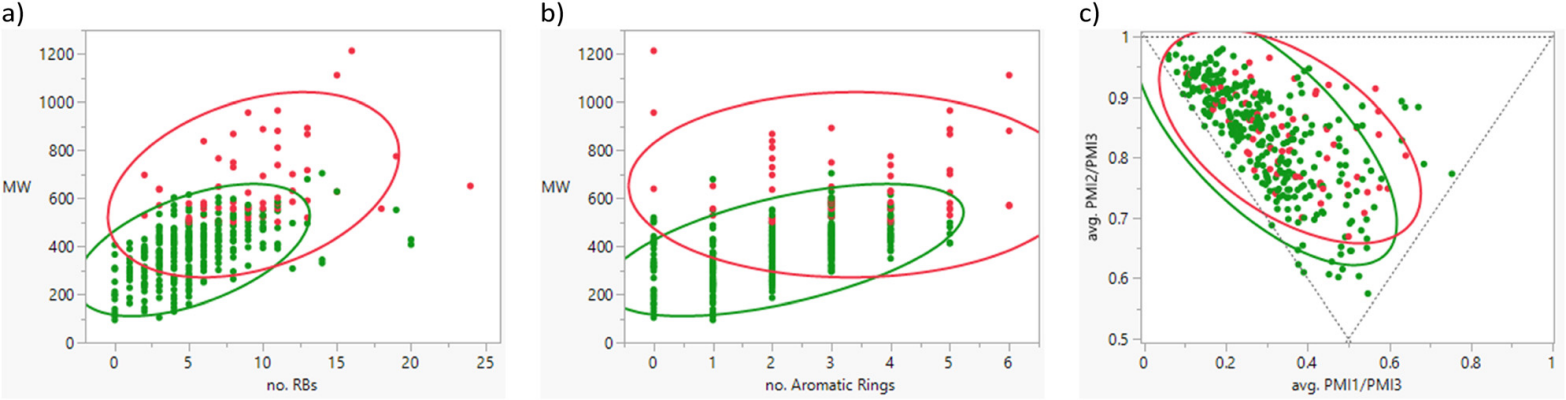
Correlations of a) rotatable bond count *vs.* MWt; b) aromatic ring count *vs.* MWt; c) average PMI1/PMI3 *vs.* PMI2/PMI3 (PMI plot), which indicates linear (coordinates: 0,1), disc-like (0.5,0.5) and spheroid (1,1) compounds. Individual points and 95% density ellipses are shown for Lipinski passes (green) and fails (red).

The rotatable bond count distribution for the set shows a profile akin to what might have been expected (mean 6, 90th %ile 11, Table 3), despite the observed increased MWt distribution. A comparison of MWt and rotatable bond count shows that the high MWt compounds often still have lower rotatable bond counts (Fig. 3a). However, comparing Lipinski fails to passes reveals that the mean rotatable bond count distributions are significantly different between the pass and fail set (mean 5.3 and 9.4, 90th %ile 9 and 14 respectively, Fig. S2a†).

**Table 3:** Rotatable bond (RB) count, aromatic ring count and fraction of sp^3^ atom (Fsp^3^) distributions of FDA approved oral drugs from 2000–2022 (*n* = 371). Outlier points on box plots show Lipinski pass (green) and fail (red)

	RBs	Aromatic rings	Fsp^3^
	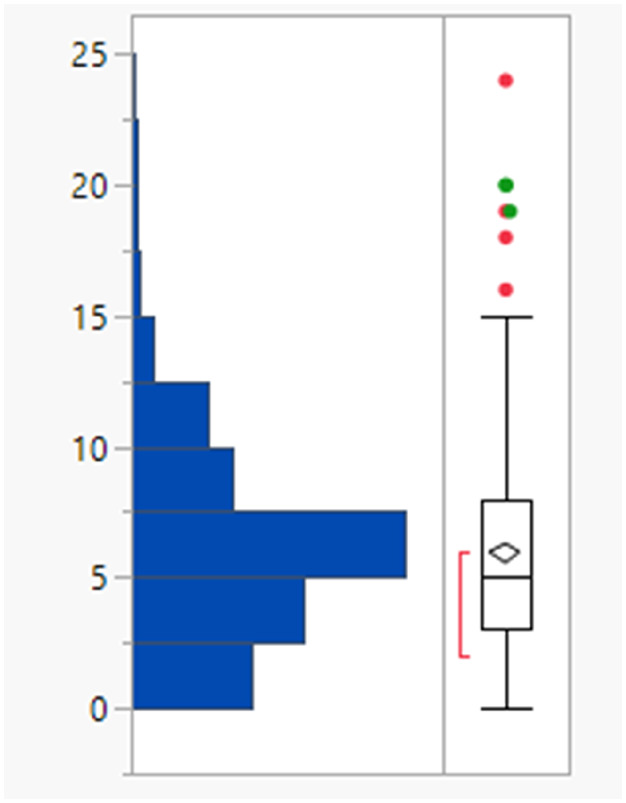	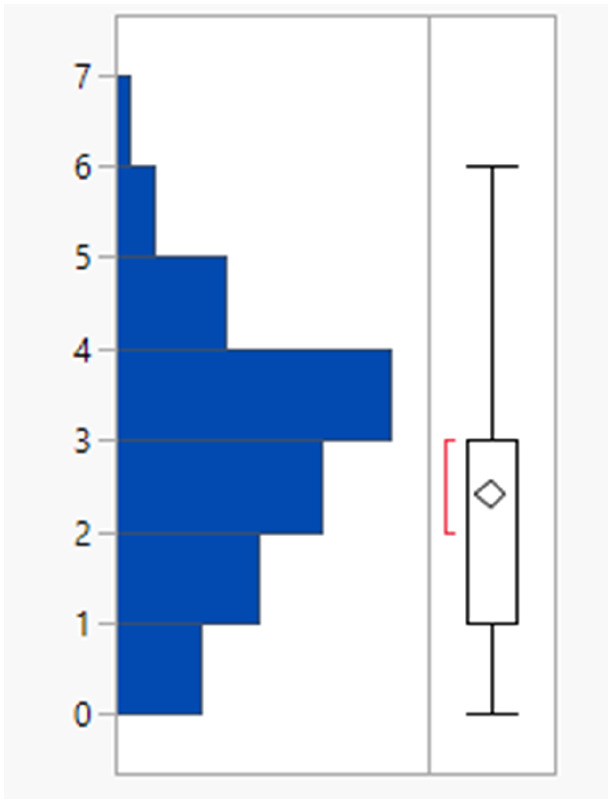	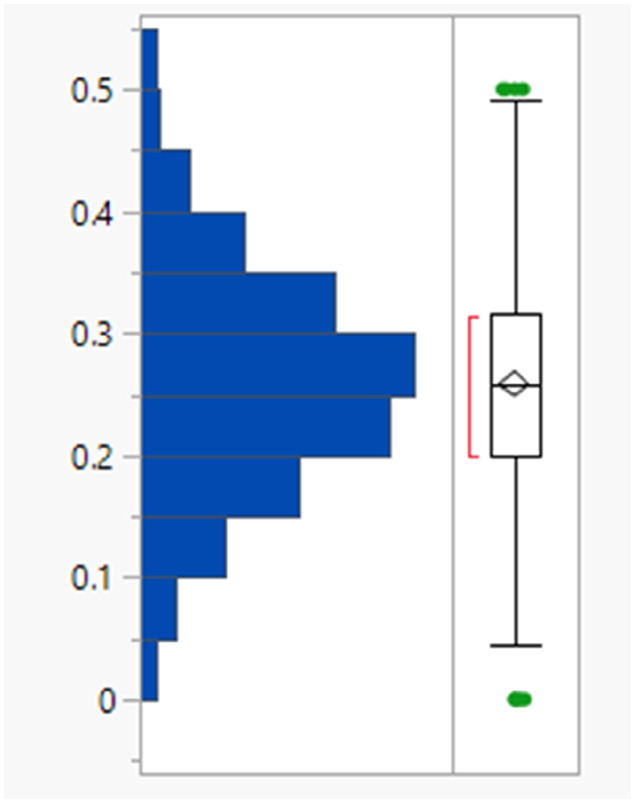
Mean (std dev)	6 (4)	2 (1)	0.26 (0.09)
50/75/90 %ile	5/8/11	3/3/4	0.26/0.32/0.38

The drug set has a mean aromatic ring count of 2, median 3 with a reasonably marked drop off above 3 (90th %ile 4). This suggests that restricting the number of aromatic rings during optimisation is likely to be worthwhile. Lipinski fails had a slightly higher aromatic ring count distribution (mean = 2 for passes, 3 for fails) but this difference appears less marked than the differences in MWt (Fig. 3b and S2b†).

The compounds span a wide range of Fsp^3^ (mean = 0.26); the distributions of the passes and fails are very similar (Fig. S2c†). Fsp^3^ is at best only weakly related to 3-dimensionality, but a further analysis using principal moments of inertia (PMI) shows that the drugs generally possess predominantly rod-like or disc-like character with very few that are spheroid (Fig. 3c). The passes and fails distribute similarly. This implies that there is no particular advantage to increased 3-dimensionality in drug discovery despite the belief that this could be advantageous and that this is not an important consideration for beyond rule of 5 design. There is no major temporal change in aromatic ring count or Fsp^3^, while there is a small change towards decreased 3-dimensionality in the latter decade (statistically significant but not meaningful), despite publications encouraging the converse (Fig. S3†).^19,20^ Any firm conclusions from such an analysis are of course dependent on the approach to conformer generation and sampling and so should be treated with caution.

Macrocyclic compounds have attracted specific interest as beyond rule of 5 compounds primarily because of their restricted degrees of freedom, which can impact greater permeability and potentially metabolic stability relative to non-macrocycles of equivalent size. There were 10 macrocycles (containing a ≥12-membered ring) in the dataset, of which 9 failed Lipinski criteria.

The macrocycles had statistically significant higher MWt, HBD, HBA and aromatic ring count than the rest of the dataset (Table 4). The difference in the distributions was smaller for HBD. Distributions of clog *P*, RBs and aromatic ring count were not significantly different. This suggests that macrocycles are a class of structures that allow greater chance of achieving oral drugs with high MWt by reducing degrees of freedom, but that lipophilicity and hydrogen bonding still need to be controlled. The sample size is of course smaller than ideal.

**Table 4:** Physicochemical property distributions of macrocycles (containing ≥12-membered ring, *n* = 10) and comparisons to the rest of the dataset

	MWt	clog *P*	HBDs
	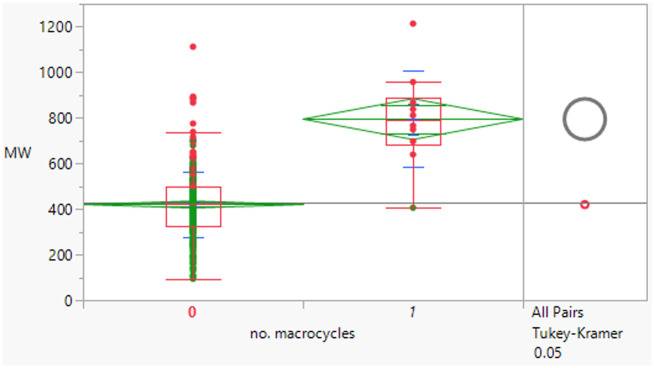	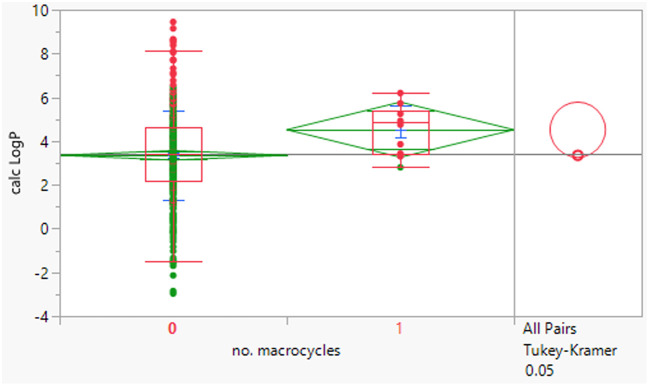	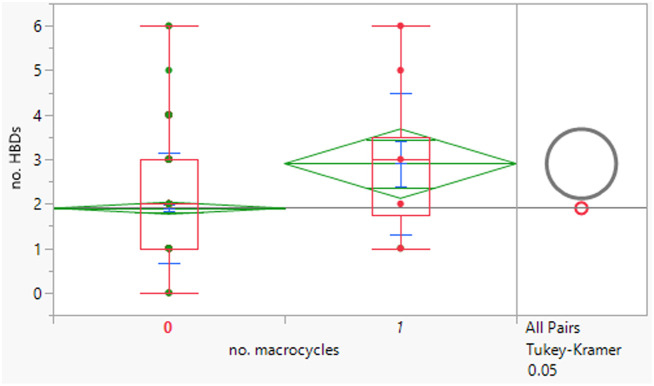
Mean (std dev)	795 (210)	4.5 (1.1)	3 (2)
50/75/90 %ile	789/891/1188	4.8/5.4/6.2	3/4/6
	HBAs	RBs	Aromatic ring count
	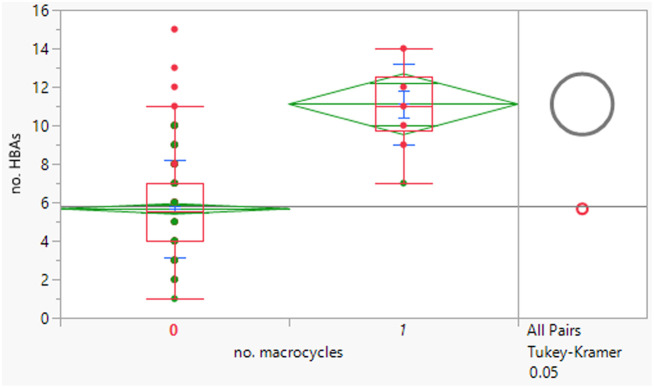	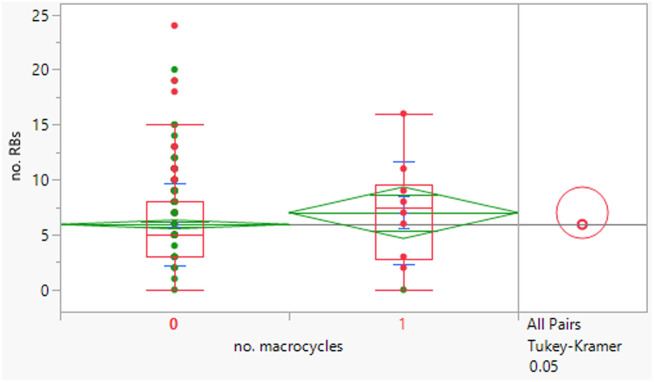	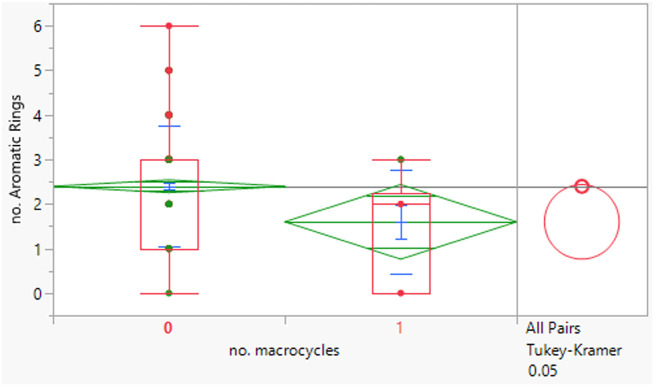
Mean (std dev)	11 (2)	7 (5)	2 (1)
50/75/90 %ile	11/13/14	8/10/16	2/2/3

## Conclusions

The trends in the data that are described here are mostly consistent with those described previously^12,13^ on partially overlapping datasets and the same observations towards increased molecular weight, for example, persist. However, we emphasise that working with smaller compounds is still beneficial and control of molecular size should not be abandoned altogether. It is likely always preferable to control physicochemical properties in optimisation to achieve attractive ADMET properties in candidate compounds. This is supported by the observation that the majority of marketed drugs continue to lie within the expected ranges for MWt, clog *P*, HBD and HBA and are Lipinski compliant. Nevertheless, a significant number of approved oral drugs lie outside of this space, demonstrating that such considerations should not be applied as rigid constraints on compound design. In cases where the biological target/mechanism necessitates non-compliant molecules, it is reassuring to see that oral drugs continue to be discovered that lie outside of this space, although to do so is always likely to be more challenging. It should be emphasised that analyses of this type, although widely influential, suffer from the impossibility of defining a suitable comparative set of compounds (non-drugs) by with which to compare.

It could be logically argued that highly potent compounds may tolerate compromised ADME properties that may result from sub-optimal physicochemical properties and hence that improving potency during optimisation could be done at the expense of drug-like properties. However, for many targets that are not amenable to high affinity ligands, it might be expected that further compromise would be required to achieve exquisitely high potency. We have not considered this in this analysis because of the difficulties with comparing *in vitro* potency across different targets, which may use different assays and translate differently to *in vivo*.

The findings reported here suggest that control of hydrogen bonding and, to a lesser extent, lipophilicity is more important than molecular size in achieving oral drugs, hence targets requiring larger molecules may be tractable provided that hydrogen bonding and lipophilicity can be controlled. This is in line with findings reported previously.^12^ The observations suggest that restricting conformational freedom, as crudely assessed by rotatable bond counts, can be useful in achieving oral drug-likeness for larger compounds. There is no clear indication that increased 3-dimensionality is beneficial. Further understanding of the properties of compounds that impart oral drug-likeness outside of classical ranges of physicochemical descriptors is required to further increase the probability of success in this region, which will be required as part of the endeavour to expand the range of tractable therapeutic targets. We would postulate that further understanding of substructural classes and their associated molecular shapes and conformational ensembles will be required to achieve this goal and such considerations are more complex than simple molecular descriptors can inform. Our findings are consistent with the concept that macrocycles are beneficial in this regard.^23,24^ Identification of further molecular sub-classes that impart similar benefits would be highly desirable.

## Computational methods

To investigate the properties of the FDA-approved drugs, a selection of descriptors available in the RDKit package were computed, and trends were investigated in SAS JMP ver. 15.2.0. The available descriptors fall into five broad categories – bond counts, atom counts, surface area, 3D shape, and ‘other’. For simplicity, only descriptors discussed in the main text are mentioned here, but a full list of computed properties, along with code and SMILES for the 2000–2022 FDA approved drugs are provided in the accompanying GitHub repository.^25^

To compute the 3D descriptors, it is important to sample a representative set of molecule geometries. A conformer ensemble was therefore generated using RDKit's experimental torsion with the “basic” knowledge distance geometry (ETKDG) algorithm, with a RMS pruning threshold of 0.1 Å, 1000 maximum attempts at embedding, random initial coordinates, consideration of small ring torsions and a random seed for reproducibility.^26,27^ The number of conformers to generate was selected using a set of rules proposed by Ebejer *et al.* derived from the number of rotatable bonds: 50 conformers for ≤7 rotatable bonds, 200 conformers for 8 ≤ rotatable bonds ≤ 12, and 300 conformers for >12 rotatable bonds.^28^ Each conformer was optimised, and the energy calculated, using the MMFF94 force field.^29^ For a property (*A*), the Boltzmann-average over all the generated conformers (*i*) was computed as:
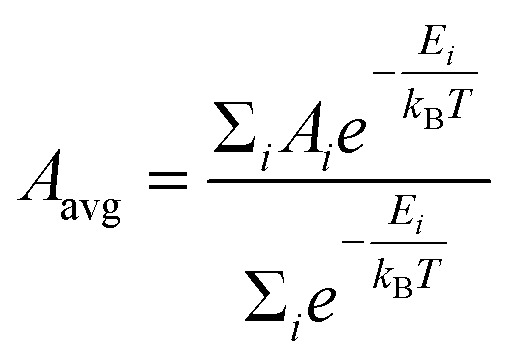
where *E*_*i*_ is the force field energy of conformer *i*, *k*_B_ is the Boltzmann constant, and *T* (=300 K) is the temperature.

### Bond count properties

The bond count descriptors included in this study are mostly available natively in RDKit from the rdMolDescriptors module. The number of rotatable bonds (CalcNumRotatableBonds) is obtained by counting the number of matches to a smarts pattern (either loose or strict) defining a rotatable bond. The aromatic ring count (CalcNumAromaticRings) is obtained by looping over the bonds within a ring and checking for aromaticity. The stereocentre count (CalcNumAtomStereoCenters) is obtained by counting the number of atoms where chirality is possible. The small ring count (CalcNumRings) is derived from the RingInfo functionality in RDKit, again derived by counting bonds. Whether the molecule is macrocyclic is not directly implemented within RDKit but can be determined by a substructure search for the SMARTS pattern “r-12” (ring of 12 or more atoms).^30^

### Atom counts

With some manipulation, the atom class can be used to obtain the number of non-carbon atoms (atom symbol not equal to C), the number of aromatic atoms (count the number of atoms that pass GetIsAromatic()), and the number of sp^3^ atoms (count of the number of atoms where GetHybridization() == HybridizationType.SP3).

### 3D shape

The first three principal moments of inertia (PMI) are a measure of the rotational dynamics of a molecule, and can be calculated computationally, or derived experimentally from IR or microwave spectra. The PMIs indicate the degree of rod-, disc- or sphere-shape a molecule has.^31^ For a specified axis, the moment of inertia, *I*, is defined as:*I* = Σ_*i*_*m*_*i*_*r*^2^_*i*_where *m*_*i*_ is the mass of atom *i*, and *r*_*i*_ is the perpendicular distance from the principles axes (with *I*_1_ ≤ *I*_2_ ≤ *I*_3_). Several descriptors of shape can be derived from PMI.^32^ The normalised principal moment ratios were proposed by Sauer and Schwarz^33^ as a crude measure of shape, independent of molecular size, obtained by dividing the two smaller moments by the largest.

## Data availability

The raw dataset is provided as a .csv file. All analysis methods are provided in the manuscript and ESI.†

## Author contributions

RP and DJC generated the RDKit descriptors, SP, HAS-G, HLS, JT and KLW compiled and validated the dataset, KF, SP, JT and MJW carried out the data analysis, and MJW wrote the manuscript. All authors made a roughly equal contribution to the work and the order of the author list does not necessarily reflect the level of contribution. Any author may list themselves as a first author if they wish.

## Conflicts of interest

There are no conflicts to declare.

## Supplementary Material

MD-015-D4MD00160E-s001
