# Supplementary material for: An analysis of the physicochemical properties of oral drugs from 2000 to 2022
Source: RSC Med Chem. 2024 Jul 22;15(9):3125–32. doi: 10.1039/d4md00160e (PMC11411612; doi:10.1039/d4md00160e)

Electronic Supplementary Material (ESI) for RSC Medicinal Chemistry.
This journal is © The Royal Society of Chemistry 2024

Supplementary information

An analysis of the physicochemical properties of oral drugs from 2000 to 2022

Table S1 – Cross-correlation coefficients (r^2) of Lipinski descriptors for the dataset. n = 372

	HBAs	HBDs	clogP
MWt	0.69	0.27	0.65
clogP	0.12	-0.14	
HBDs	0.27		

Table S2 – Analysis of mean clogP values for neutral and charged compounds

Level	N	Mean	Std Dev	Std Err Mean	Lower 95%	Upper 95%
Neutral	149	3.4301221	2.1490323	0.1760556	3.0822147	3.7780295
Charged	229	3.4387821	2.0254492	0.1338455	3.1750499	3.7025142

Figure S1 – Lipinski fails (blue) and passes (red) over time a) Mosaic plot by decade; b) Table of counts; c) Mosaic plot by year; d) Histogram by year.

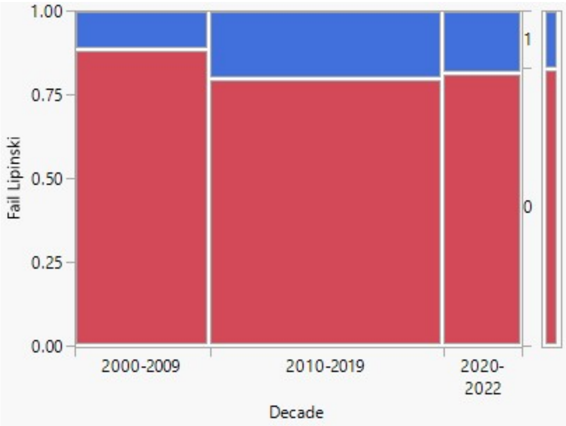

a)

b)

Count	Pass	Fail	Total
2000-2009	99 (88%)	13 (12%)	112
2010-2019	155 (80%)	39 (20%)	194
2020-2022	53 (82%)	12 (18%)	65
Total	307	64	371

c)

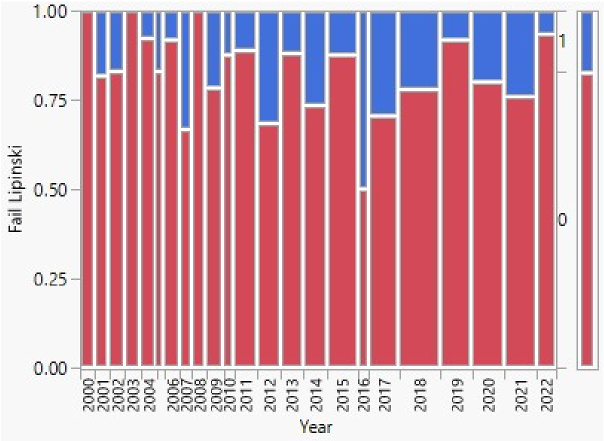

d)

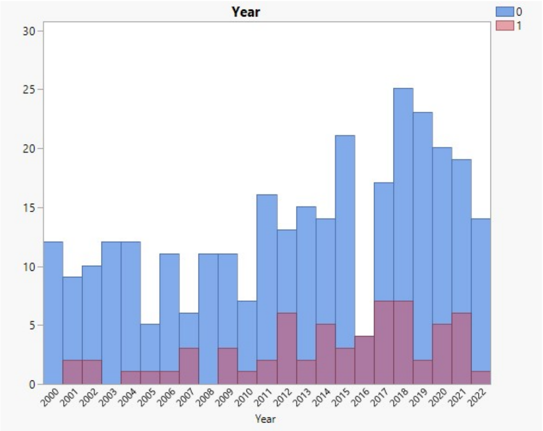

Table S3

Mean (std dev) — MWt — clogP

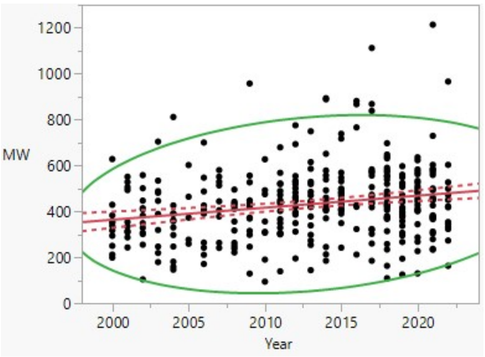

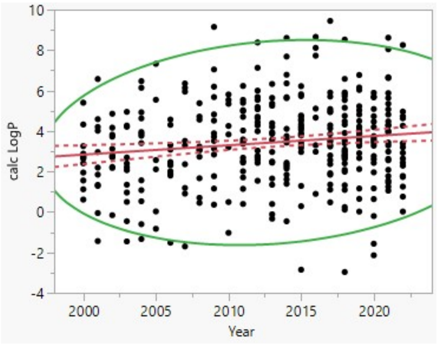

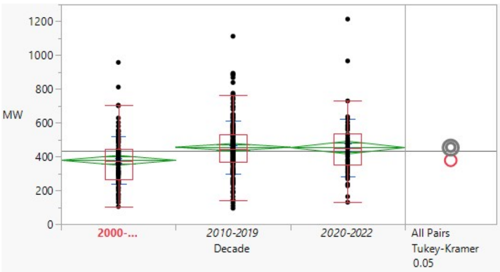

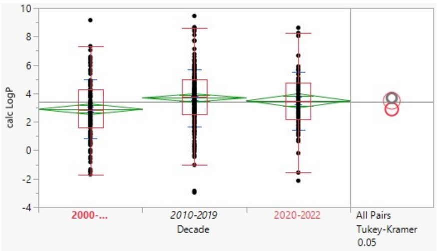

Mean (std dev)	MWt	clogP
2000-2009	378 (139)*	2.9 (2.1)**
2010-2019	455 (156)	3.7 (2.0)
2020-2022	453 (169)	3.5 (2.0)

HBDs — HBAs

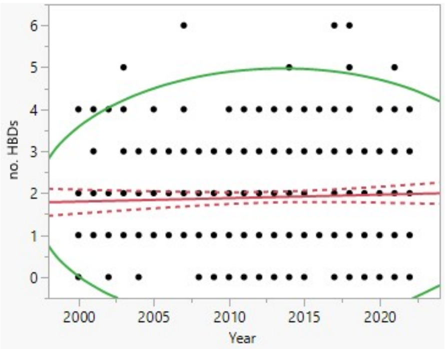

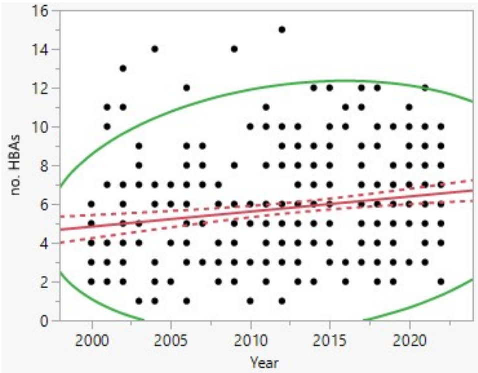

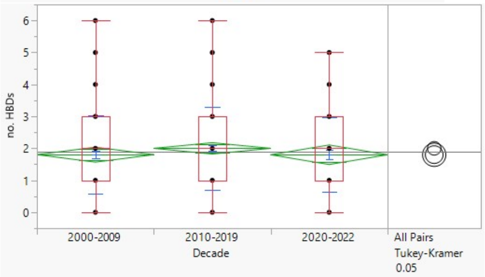

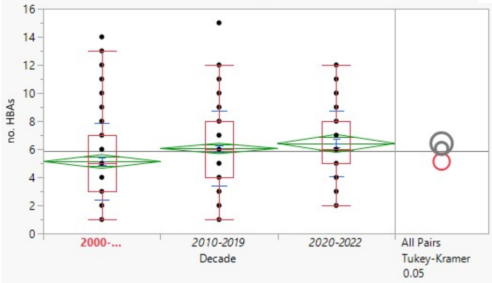

	HBDs	HBAs
2000-2009	2 (1)	5 (3)*
2010-2019	2 (1)	6 (3)
2020-2022	2 (1)	6 (2)

*Value statistically significantly different (Tukey-Kramer method), ** significantly different from 2010-2019 but not 2020-2022.

Figure S2 – Comparison of a) rotatable bond count; b) aromatic ring count; c) Fsp^3 between the Lipinski passes and fails.

a)

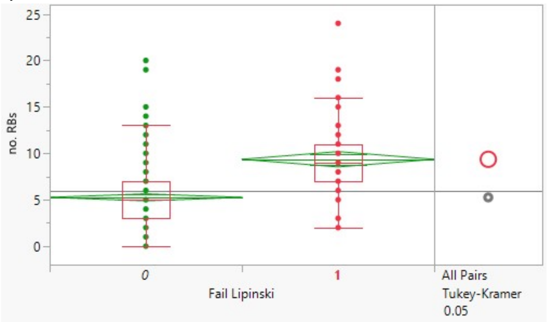

b)

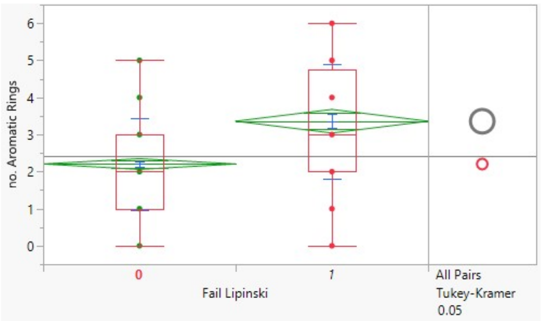

c)

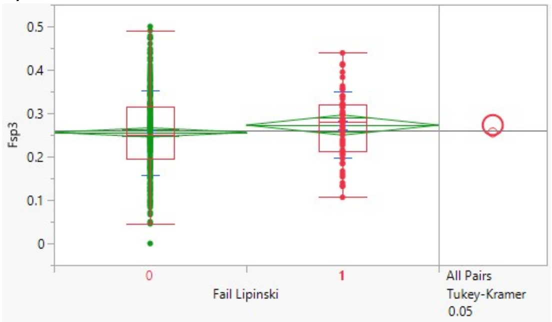

Figure S3 – Variation of a) aromatic ring count; b) Fsp^3 by decade.

a)

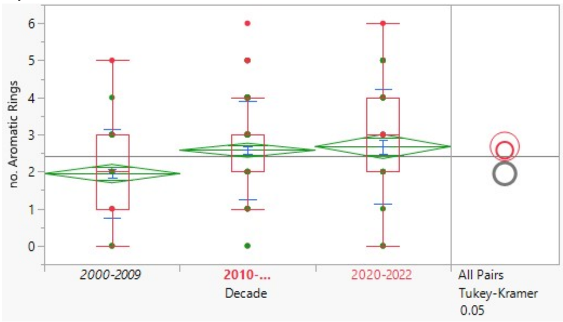

b)

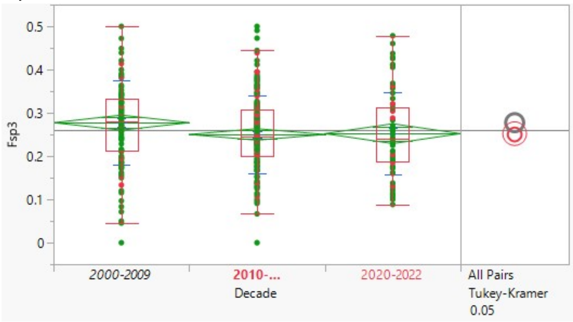

Supplement: MD-015-D4MD00160E-s001 [file MD-015-D4MD00160E-s001.pdf]
